# Beyond the Breaking Point: A Unique Case Report of a Penile Fracture With a Distal Urethral Injury

**DOI:** 10.7759/cureus.46268

**Published:** 2023-09-30

**Authors:** Mohamed Javid, Ananda Kumar Ilangovan, Sudhakaran Selvaraj, Ramesh Ganapathy, Srikala Prasad

**Affiliations:** 1 Urology, Chengalpattu Medical College, Chengalpattu, IND

**Keywords:** urethral rupture, urological emergency, blunt trauma, urethral injury, penile fracture

## Abstract

A penile fracture is a rare urological emergency, often resulting from blunt trauma to the erect penis. This case report describes a 30-year-old male who presented with penile swelling during sexual intercourse, raising suspicion of a penile fracture. The patient underwent surgical exploration, revealing a rent in the tunica albuginea and an additional laceration in the corpus spongiosum with the Foley catheter exposed. The lacerations were closed using Vicryl sutures over a Foley catheter. The patient experienced an uneventful postoperative course, and follow-up assessments showed satisfactory healing of the urethra. This case underlines the need for clinicians to consider the possibility of urethral involvement in cases of penile fracture, as timely surgical intervention can prevent long-term sequelae such as erectile dysfunction and urethral strictures. By sharing this case, we hope to further emphasize the need for vigilance and swift action when faced with potential penile fractures in clinical practice.

## Introduction

A penile fracture is an uncommon but significant urological emergency, with an incidence of around 1;1,75,000 in the U.S. population [1,2]. It is still underreported in developing countries [2]. It usually results from blunt trauma to the erect penis, which is usually caused by vigorous sexual intercourse or masturbation, and presents with sudden penile pain, swelling, and deformity [1,3,4]. Although penile fractures are a well-described entity, there is limited literature on cases with urethral involvement. In this report, we present a case of penile fracture with urethral involvement and provide a comprehensive discussion of its clinical presentation, management, and outcomes. By sharing our experience, we aim to highlight the importance of the early diagnosis and proper management of this condition, particularly in cases with urethral involvement.

## Case presentation

A 30-year-old male presented to the emergency department with penile swelling and mild blood at the meatus. Initially, the patient provided a false history, claiming that he had lost consciousness and fell, which directed the initial evaluation toward potential causes of loss of consciousness rather than focusing the attention on the penile swelling. Upon further inquiry and in privacy, he revealed that he experienced sudden penile swelling during sexual intercourse, accompanied by a sudden loss of tumescence. He also reported a history of hematuria. On examination, the penis was swollen, and careful palpation revealed an irregularity at the 7 o'clock position around 2 cm proximal to the coronal sulcus, which was clinically suspected as a penile fracture.

**Figure 1:**
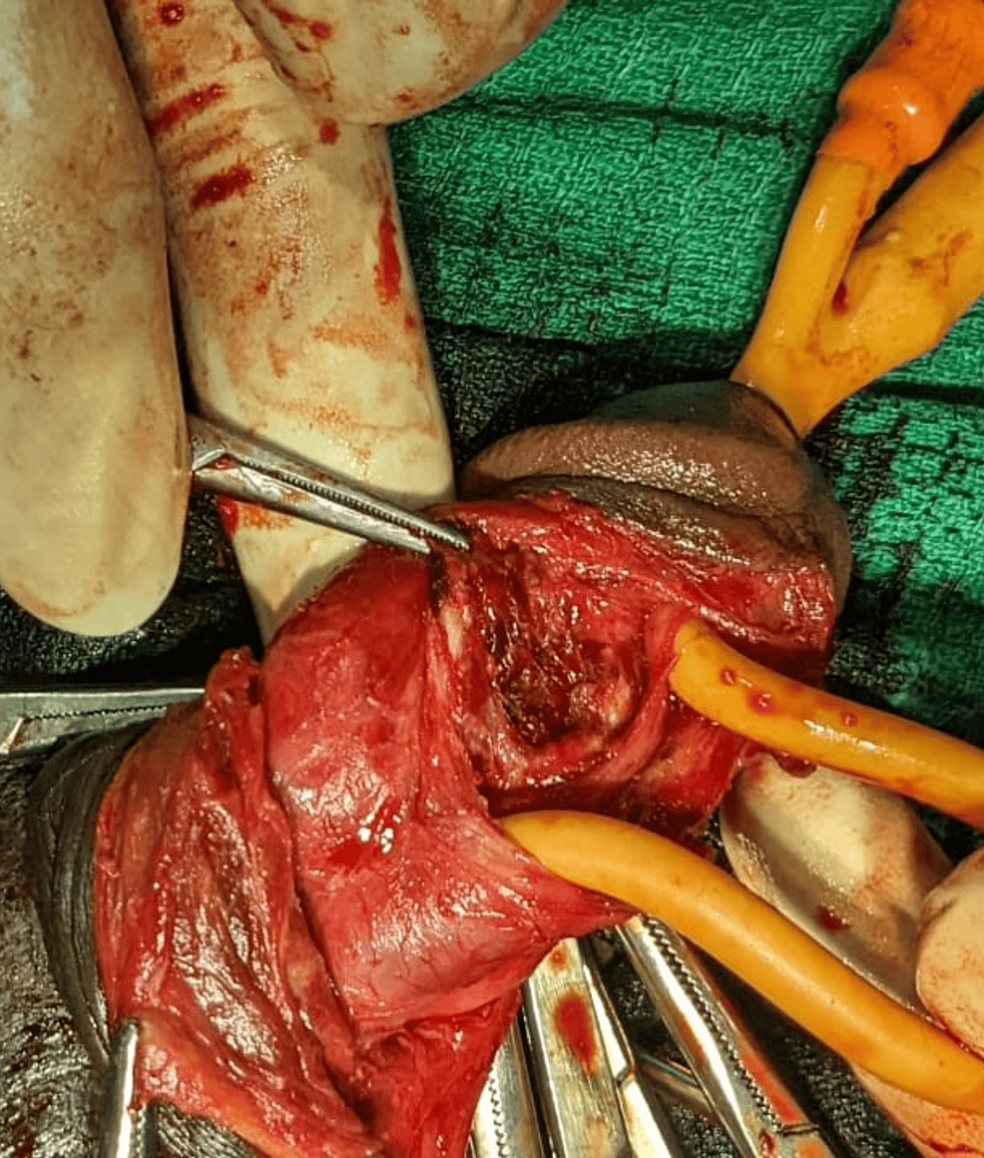
Lacereated tunica albuginea and lacerated corpus spongiosum with the Foley catheter exposed

Cystoscopy (rigid) subsequently confirmed the status of the urethral mucosa and bladder before exploration. Irregular urethral mucosa was observed at the 6 o'clock position around 2-3 cm from the external urethral meatus and a 16 Fr Foley catheter was placed. A circumcoronal degloving incision was made 1 cm proximal to the coronal sulcus, revealing a 3 cm laceration in the tunica albuginea at the 7 o'clock position and another 2 cm laceration in the corpus spongiosum at the 6 o'clock position, with the Foley catheter exposed (Figure 1).

**Figure 2:**
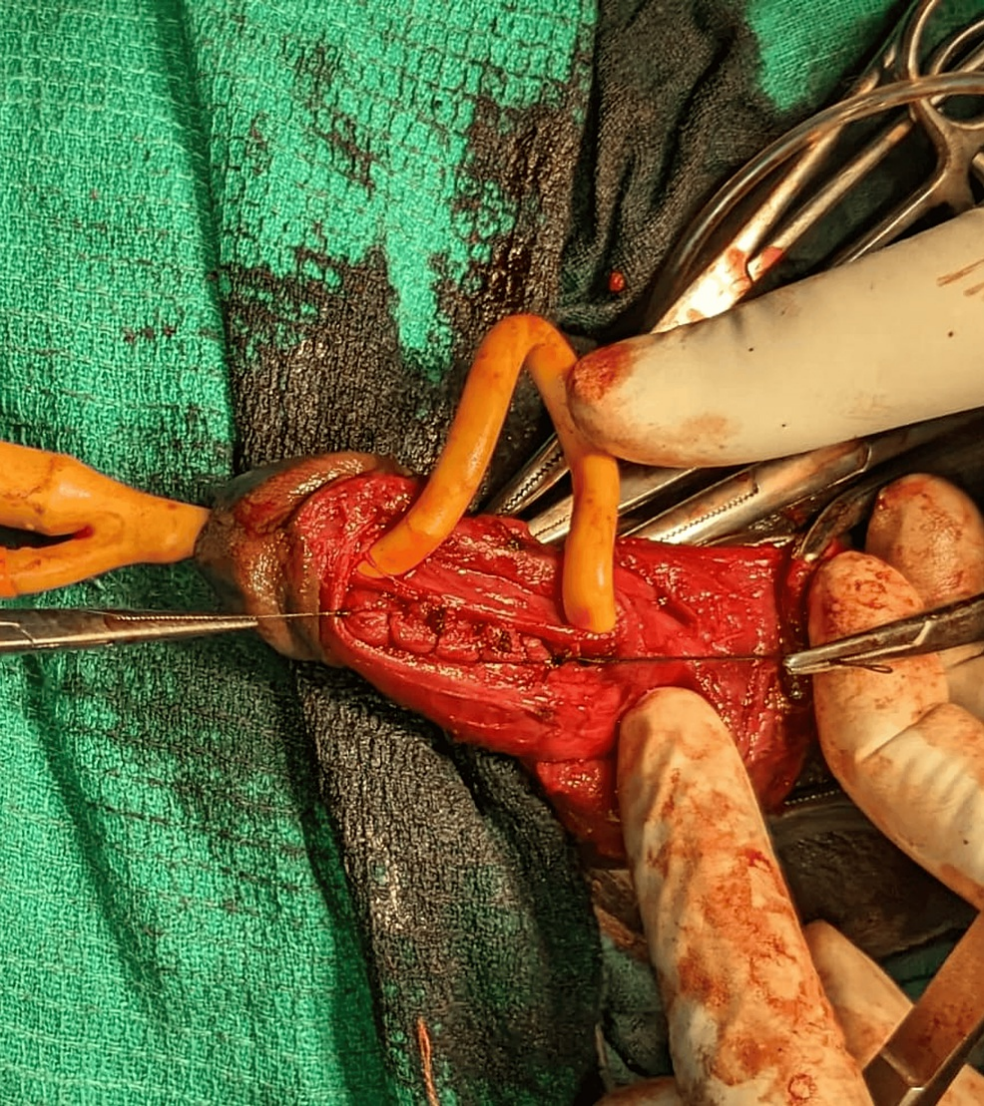
Tunica albuginea post-repair

Both lacerations were closed separately with 3-0 Vicryl sutures over the 16 Fr Foley catheter (Figure 2).

The postoperative period was uneventful, and the Foley catheter was removed after three weeks. Follow-up cystoscopy at three months showed good healing of the urethra, with no stricture formation. The patient indicated satisfactory erectile function for sexual activity, without experiencing pain or discomfort during coitus. Additionally, the individual demonstrated good urinary voiding, as evidenced by a normal uroflowmetry assessment.

## Discussion

Penile fractures are rare but serious urological emergencies that require timely diagnosis and appropriate early management to minimize long-term complications [4]. The most common cause of penile fractures is blunt trauma during sexual activity, which can cause a sudden and forceful bending of the erect penis, resulting in the rupture of the tunica albuginea, the fibrous covering of the corpora cavernosa [1]. The resulting loss of erection is often accompanied by a characteristic popping sound, severe pain, and swelling of the penis [5].

The diagnosis of penile fracture is based on a careful clinical history and examination. Patients usually present with a sudden loss of erection and severe pain, often accompanied by swelling and bruising of the penis [5]. However, as in this case report, patients may not provide a full history initially, which can lead to a delay in diagnosis. It is therefore essential for healthcare providers to take a detailed history from the patient, with adequate privacy and sensitivity, to ensure that all relevant information is disclosed. Physical examination may reveal a deformity or buckling of the penis, and palpation may elicit crepitus or a crunching sensation, indicating a fracture of the tunica albuginea. When there is ambiguity, investigations, such as ultrasound or MRI, may be used to confirm the diagnosis and assess the extent of the injury [4].

Another important consideration is the possibility of associated urethral injury. Several studies have reported an incidence of urethral injury ranging from 10% to 20% in patients with penile fractures [5,6]. A high index of suspicion is necessary, especially when there is a history of hematuria, as seen in this patient [5]. An associated urethral injury can have long-term implications, including urethral stricture, which can cause urinary symptoms and require further intervention. Hence, careful follow-up is important for such patients [5,7].

The management of penile fractures involves a prompt surgical intervention to repair the tunica albuginea and any associated urethral injuries. The surgical approach may vary depending on the location and severity of the fracture and associated injuries. In general, open surgical repair remains the standard of care for most cases of penile fracture, especially those with associated urethral injury. The choice of incision can be decided based on the case scenario. A longitudinal incision can be made over the suspected injury site if a small laceration is suspected, whereas a circumcoronal incision might be needed if the injury needs a formal exploration or if a urethral injury is suspected [4]. In most instances, penile fractures involve the right side and the ventrolateral part of the proximal third of the penis; however, the case described here exhibits a noteworthy deviation. Though the fracture appeared on the right side, it was predominantly situated in the distal area of the penis, illustrating the heterogeneity of penile fracture cases [8].

Complications of penile fractures can be significant and long-lasting even after surgery. Erectile dysfunction is a common complication and can result from damage to the neurovascular bundle or fibrosis of the corpora cavernosa [9,10]. Penile curvature or deformity can also occur, which can affect sexual function and lead to psychological distress. Urethral stricture, secondary to urethral injury, is another potential complication that can cause urinary symptoms and require further intervention [9,10].

## Conclusions

Prompt diagnosis of penile fractures and appropriate management are crucial in optimizing outcomes. The sensitivity of the topic and the potential variability in penile fracture manifestations underscore the need for a detailed history to ensure all relevant information is disclosed. With early surgical intervention, the risk of long-term complications, such as erectile dysfunction and urethral stricture, can be minimized.
